# Splenorenal shunt for reconstruction of the gastric and splenic venous drainage during pancreatoduodenectomy with resection of the portal venous confluence

**DOI:** 10.1007/s00423-021-02318-2

**Published:** 2021-10-07

**Authors:** Mohammed Al-Saeedi, Leonie Frank-Moldzio, Pietro Contin, Philipp Mayer, Martin Loos, Thomas Schmidt, Martin Schneider, Beat P. Müller-Stich, Christoph Berchtold, Arianeb Mehrabi, Thilo Hackert, Markus W. Büchler, Oliver Strobel

**Affiliations:** 1 Department of General, Visceral and Transplantation Surgery, University Hospital Heidelberg, Im Neuenheimer Feld 420, 69120 Heidelberg, Germany; 2 Department of Diagnostic and Interventional Radiology, University Hospital Heidelberg, Heidelberg, Germany; 3 Present Address: Division of Visceral Surgery, Department of General Surgery, Medical University of Vienna, Währinger Gürtel 18-20 - AKH Wien, Ebene 07, 1090 Vienna, Austria

**Keywords:** Splenorenal shunt, Pancreatoduodenectomy, Splenic vein ligation, Venous drainage

## Abstract

**Background:**

Resection of the portal venous confluence is frequently necessary for radical resection during pancreatoduodenectomy for cancer. However, ligation of the splenic vein can cause serious postoperative complications such as gastric/splenic venous congestion and left-sided portal hypertension. A splenorenal shunt (SRS) can maintain gastric and splenic venous drainage and mitigate these complications.

**Purpose:**

This study describes the surgical technique, postoperative course, and surgical outcomes of SRS after pancreatoduodenectomy.

**Methods:**

Ten patients who underwent pancreatoduodenectomy and SRS between September 2017 and April 2019 were evaluated. After resection an end-to-side anastomosis between the splenic vein and the left renal vein was performed. Postoperative shunt patency, splenic volume, and any SRS-related complications were recorded.

**Results:**

The rates of short- and long-term shunt patency were 100% and 60%, respectively. No procedure-associated complications were observed. No signs of left-sided portal hypertension, such as gastrointestinal bleeding or splenomegaly, and no gastric/splenic ischemia were observed in patients after SRS.

**Conclusion:**

SRS is a safe and effective measure to mitigate gastric congestion and left-sided portal hypertension after pancreatoduodenectomy with compromised gastric venous drainage after resection of the portal venous confluence.

## Introduction

Pancreatic cancer is characterized by poor prognosis due to late diagnosis and aggressive biology with early local invasion and high potential for systemic metastasis [1]. Radical and high-quality surgical resection in combination with systemic chemotherapy remains the only curative treatment option for this aggressive disease. In case of vascular involvement, extended resection with vascular reconstruction is frequently necessary even after neoadjuvant therapy [1–4]. To achieve clear surgical margins in borderline resectable tumors, resections of the portal and superior mesenteric vein (SMV) including the portal venous confluence are often necessary [5]. Resection of the portal confluence with division of the splenic vein (SV) usually allows for tension-free venous anastomosis between the portal vein (PV) and SMV [5]. When the SV is ligated during pancreatoduodenectomy with resection of the portal venous confluence, venous blood from the stomach and spleen can frequently drain via collateral veins including the left gastric vein (also called coronary vein) dependent on venous anatomy. Several investigators have reported a successful surgical combination of SV ligation and collateral vein preservation during pancreatoduodenectomy [6–8]. However, even after preservation of such collateral veins, several serious complications including left-sided portal hypertension, splenomegaly, thrombocytopenia, and gastrointestinal bleeding have been reported [9–11].

In the mid-twentieth century, the portosystemic shunt was introduced as a promising treatment for gastroesophageal variceal hemorrhage in patients with cirrhosis and extrahepatic portal hypertension [12]. Since then, several studies have reported long-term shunt patency and a reduction of hemorrhages following shunt operations [13, 14]. The distal splenorenal shunt (SRS) is one option of a portosystemic shunt that allows gastric and splenic venous drainage via the left renal vein into the systemic circulation [15–17]. Recently, it has been shown that performing SRS during pancreatoduodenectomy may reduce postoperative severe complications, such as left-sided portal hypertension [17, 18]. In the same time period, the procedure has been developed and implemented in our center for patients with signs of venous congestion after portal venous resection. In this study, we aim to report our experience, illustrating the surgical technique, postoperative course, and surgical outcomes of patients who underwent SRS during pancreatoduodenectomy.

## Material and methods

### Study design

The observational study is based on a retrospective analysis of clinical data prospectively collected in an institutional database of consecutive pancreatic operations. This study was approved by the institutional ethics committee and follows the STROBE recommendations for observational studies [19].

### Study population

All patients who underwent pancreatoduodenectomy and SRS between September 2017 and April 2019 in our center were included. Patient demographics, baseline data, laboratory findings, and preoperative assessment were retrospectively extracted electronically from the hospital laboratory information system. All patients underwent contrast-enhanced computed tomography and/or magnetic resonance imaging to assess tumor resectability and to plan the operation. Missing information was obtained from the patients’ records.

### Surgical technique

**Fig. 1:**
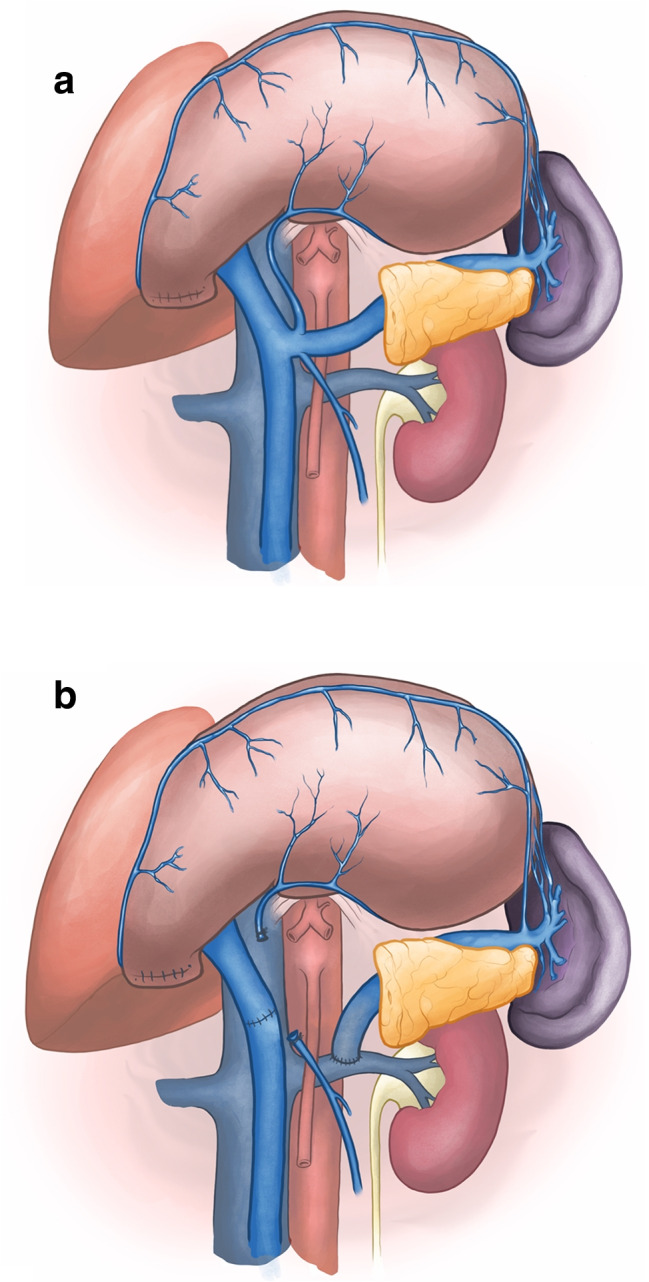
Image showing the technique **a** before and **b** after performing splenorenal shunt during pancreatoduodenectomy

All patients underwent partial or total pancreatoduodenectomy with extended lymph node dissection of the hepatic hilum, common hepatic artery, celiac trunk, and superior mesenteric artery. Our pancreatoduodenectomy technique has been described in detail elsewhere and results in exposure and easy accessibility of the left renal vein [20] (Fig. 1a).

**Fig. 2:**
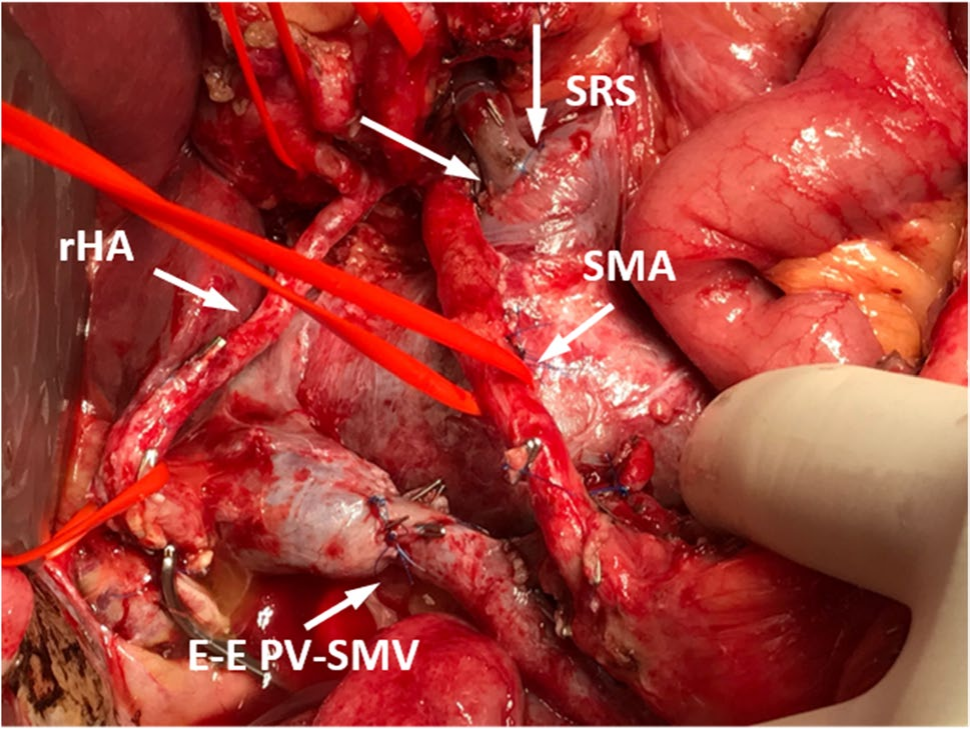
Intraoperative image showing the splenorenal shunt (SRS) after pancreatoduodenectomy. rHA, right hepatic artery; SMA, superior mesenteric artery; E-E PV-SMV, end-to-end anastomosis of portal vein and superior mesenteric vein

To perform a pancreatoduodenectomy with portal confluence resection, the pancreatic head is completely mobilized. Subsequently, the veins (PV, SMV, and SV) are clearly identified and secured with vessel loops. Next, SV is mobilized approximately 2–3 cm from the pancreas to allow for maximal exploration of the local vascular anatomy, achieve a radical resection margin, and later facilitate direct tension free venous anastomoses without interposition graft. At this time, any additional tumor infiltration, especially in the coronary vein or inferior mesenteric vein (IMV), can be assessed, as they may provide relevant venous gastric drainage in cases of SV ligation. If the IMV or coronary vein is involved, division of these veins is performed. Vascular clamps (e.g., Satinsky or bulldog clamp) [21, 22] are then applied to the SMV, PV, and SV, and the resection is completed by division of the PV, SMV, and SV, respectively. An end-to-end anastomosis between the SMV and PV is performed using a 5–0 or 6–0 monofilament non-absorbable running suture. Next, the stomach and spleen are assessed for signs of venous congestion. The decision to perform a SRS is made only if signs of venous congestion appear after the resection. In cases with congestion, usually both the coronary and inferior mesenteric vein were divided. To prepare for the anastomosis, a Satinsky vessel clamp is applied tangential to the left renal vein already exposed after the resection phase. Afterwards, the SRS is created by an end-to-side anastomosis between the SV and the left renal vein (Fig. 1b). The anastomosis is performed using a 6–0 monofilament non-absorbable running suture (Fig. 2). To assess the quality of the anastomoses, an intraoperative Doppler flowmetry is performed after each anastomosis. Prophylactic antiplatelet or other anticoagulation agents were not routinely used during the operation, unless the patient had a high risk for thrombosis [21, 22].

### Treatment-related parameters and postoperative course

Treatment-related parameters were prospectively recorded in the database including the type and extent of pancreatic resection according to the ISGPS [23], vascular resections, blood loss, and duration of the operation. Pathological parameters included pTNM tumor stage according to the 7th edition of the TNM staging manual, grading, and the resection margin (R) status.

None of the patients received antiplatelet agent due to the SRS operation. As per standard, all patients received thrombosis prophylaxis with low molecular heparin weight following surgery until discharge. Therapeutic heparinization was administrated only in patients with prior anticoagulative therapy or those with high risk of thrombosis. Any SRS-related complications and any signs of left-sided portal hypertension, such as splenomegaly, gastrointestinal bleeding, and delayed gastric emptying, were recorded from the time of operation and for the complete follow-up period. To evaluate the shunt patency after surgery and the short-term changes in spleen volume (until 3 months after the operation), all patients underwent Duplex ultrasound and/or computed tomography/magnetic resonance imaging during the follow-up period. Postoperative thrombocytopenia was defined as a platelet count of < 150/nL. The severity of postoperative complications was classified based on the Clavien–Dindo classification [24], where grade I–II morbidities were defined as minor and grade III–V morbidities as major. Mortality was defined as all-cause death occurring during the hospital stay.

### Statistical analysis

Data were analyzed using SPSS software (Version 22, IBM Corp. Released 2013. Armonk, NY). Continuous variables were presented as means with standard deviation, and categorical variables were described using frequency distributions. To analyze differences in spleen volume after SRS, a paired sample *t*-test was performed. One-year SRS patency was analyzed using the Kaplan–Meier method. *p* values ≤ 0.05 were considered significant in all analyses.

## Results

Ten patients underwent pancreatic surgery with resection of the portal confluence and simultaneous SRS. The mean age of patients was 64 ± 8 years with an equally distributed sex ratio. Most patients presented with pancreatic ductal adenocarcinoma (90%), while one patient had a neuroendocrine tumor. Three patients (30%) received neoadjuvant chemo/radiotherapy before the operation (Table 1).

**Table 1:** Clinicopathologic characteristics and perioperative data of the patients

	*n* (%) or mean ± SD
Sex	
Female	5 (50)
Male	5 (50)
Age (years)	64 ± 8
BMI (kg/m^2^)	23.2 ± 3.7
ASA classification	
I	0 (0)
II	6 (60)
III	3 (30)
IV	1 (10)
Disease	
Pancreatic ductal adenocarcinoma	9 (90)
Pancreatic neuroendocrine tumor	1 (10)
Neoadjuvant therapy	
Yes	3 (30)
Tumor localization	
Head	7 (70)
Body	3 (30)
pT stage	
1	2 (20)
2	6 (60)
3	1 (10)
4	1 (10)
pN stage	
Positive	8 (80)
Grading (*n* = 3 missing)*	
2	3 (42.9)
3	4 (57.1)
R status	
R0/CRM-	2 (20)
R0/CRM +	1(10)
R1	7 (70)

*SD* standard deviation, *BMI* body mass index, *ASA* American Society of Anesthesiologists classification, *CRM* circumferential resection margin

^*^In 3 patients, grading was not assigned after neoadjuvant chemotherapy

A pancreatoduodenectomy was performed in six (60%) patients and a spleen preserving total pancreatectomy in four (40%) patients. Five patients (50%) underwent pylorus preservation, and the remaining five patients (50%) underwent simultaneous partial gastrectomy during the operation. The mean operative time was 430.7 ± 146.2 min with an estimated mean blood loss of 1.2 ± 0.6 l (Table 2).

**Table 2:** Perioperative data of the patients

	*n* (%) or mean ± SD
Operative time (min)	430.7 ± 146.2
Estimated blood loss (l)	1.2 ± 0.6
Type of pancreatic resection	
Pancreatoduodenectomy	6 (60)
Total pancreatectomy	4 (40)
Vascular resection	
Arterial resection	4 (10)
Venous resection	10 (100)
Thrombocytopenia	
Preoperative	1 (10)
Postoperative (first week)	6 (60)
Spleen volume (ml)	
Preoperative	258.9 ± 118.5
Postoperative	251.4 ± 108.3
Splenomegaly	
Preoperative	0 (0)
Postoperative	0 (0)
Procedure-related complications	0 (0)
Left-sided portal hypertension	0 (0)
Gastrointestinal bleeding	0 (0)
Delayed gastric emptying	0 (0)
Patency of the shunt	
Short-term (first month)	10 (100)
Long-term	6 (60)
Morbidity (Clavien–Dindo)	
None	5 (50)
Minor (grade I–II)	2 (20)
Major (grade III–V)	3 (30)
Duration of hospitalization (days)	27.2 ± 23.4
In-hospital mortality	1 (7.7)

*SD* standard deviation

The operative time required for the creation of the SRS was 5–15 min, and there were no procedure-related complications (no bleeding, renal vein thrombosis, renal insufficiency). Moreover, there were no perioperative complications related to left-sided portal hypertension such as gastrointestinal bleeding and gastric/splenic ischemia. Fifty percent of our patients had postoperative complications. Two patients (20%) had minor complications, and three (30%) had major complications based on the Clavien–Dindo classification, but none of these was SRS-related complications (Table 2). One patient developed PV thrombosis 38 days after the operation and underwent re-operation with thrombectomy but died 71 days after the operation because of multiple organ failure. In this patient, the SRS remained patent until death.

**Fig. 3:**
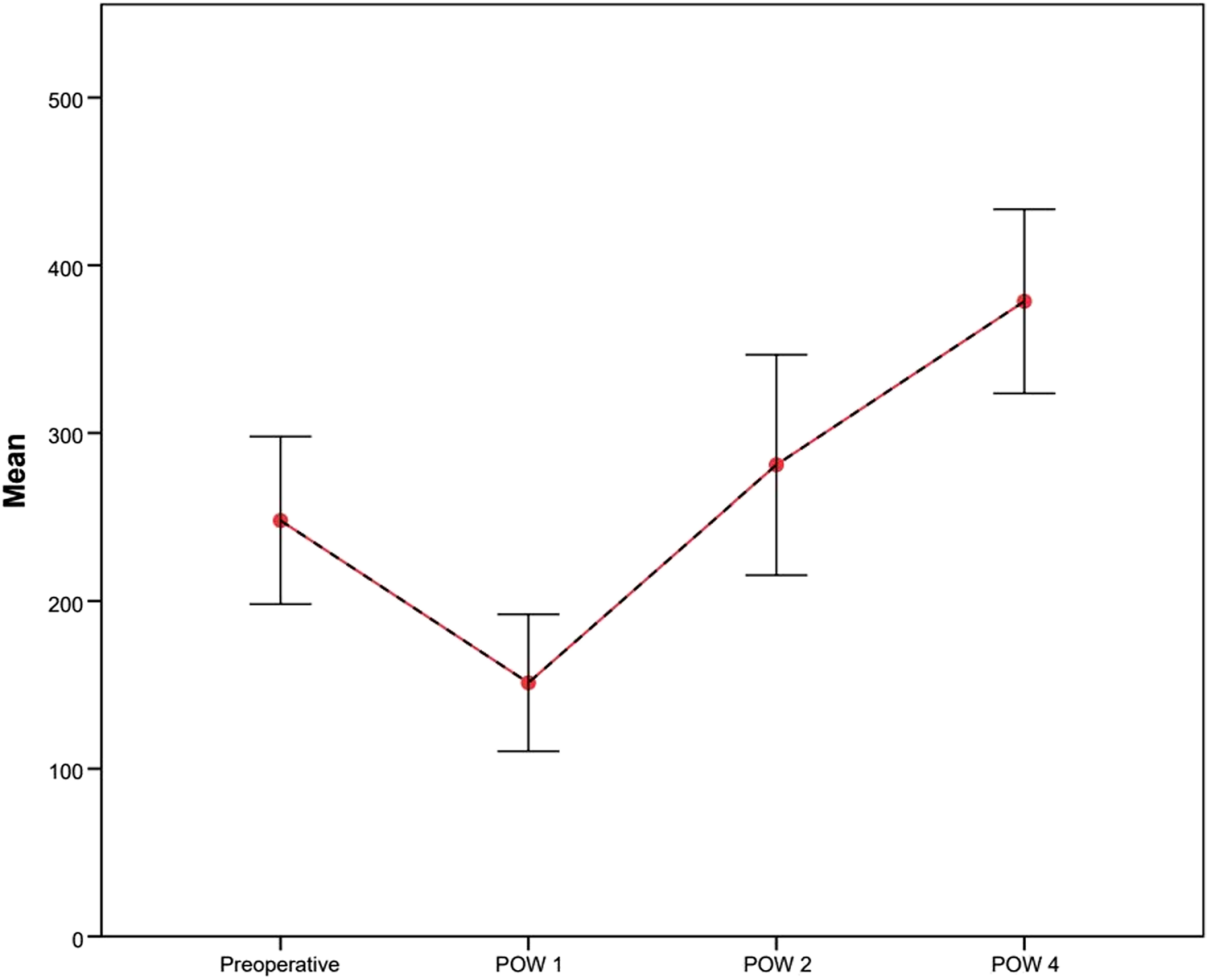
**a**, **b** Preoperative axial portal venous phase computed tomography (CT) image and coronal maximum intensity projection image showing the hypodense tumor in the pancreatic head with stenosis of the portovenous confluence (blue arrows) with consequent formation of cavernous collaterals in the liver hilum (orange arrowhead). **c**, **d** Postoperative axial and coronal portal venous phase CT images showing the patent splenorenal shunt (red arrows). Note the nutmeg appearance of the liver, probably as result of hepatic venous congestion unrelated to the splenorenal shunt

**Fig. 4:**
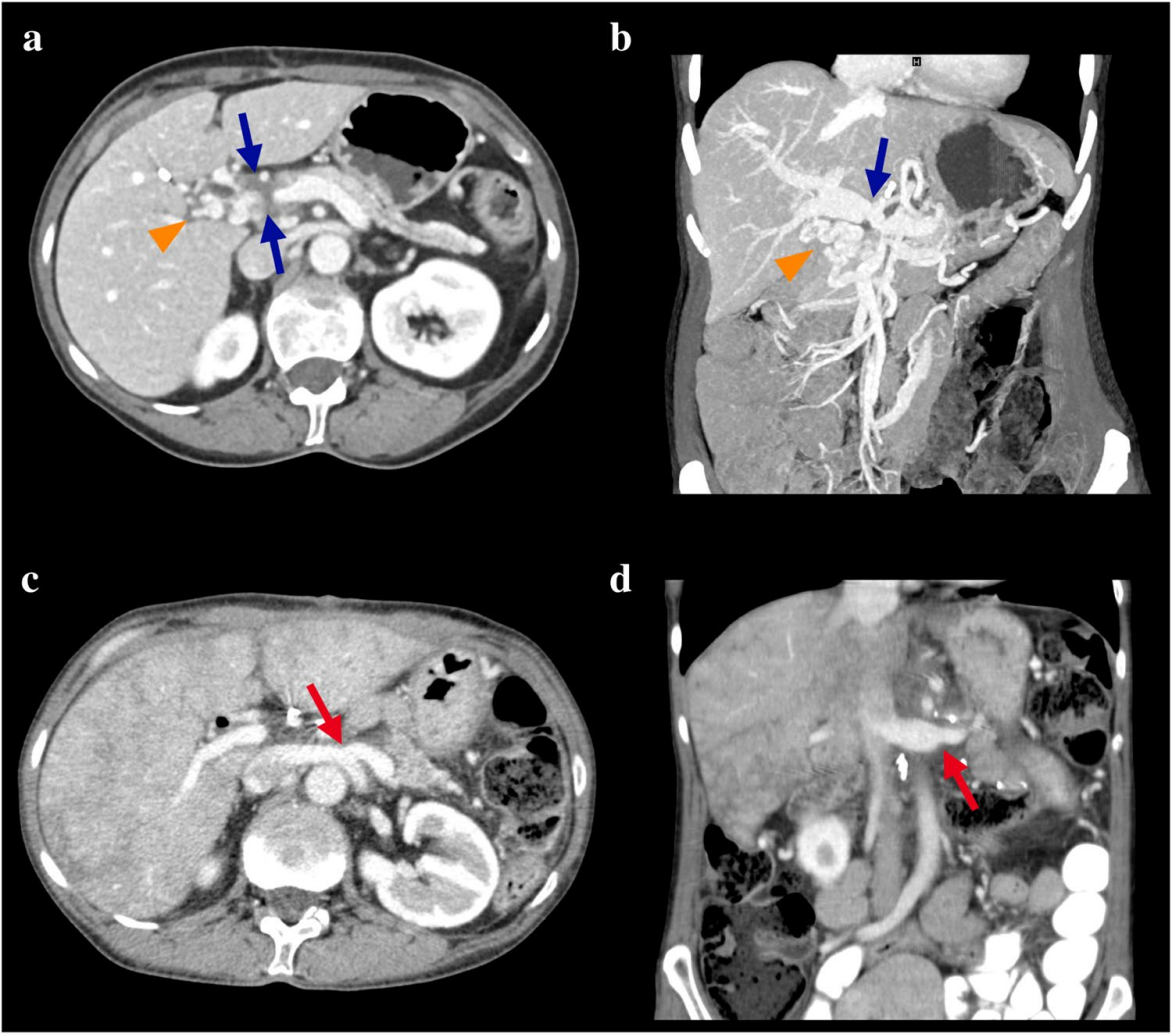
Kaplan–Meier plot showing the 1-year patency of the splenorenal shunt after the operation (1-year patency rate: 68.9%)

**Fig. 5:**
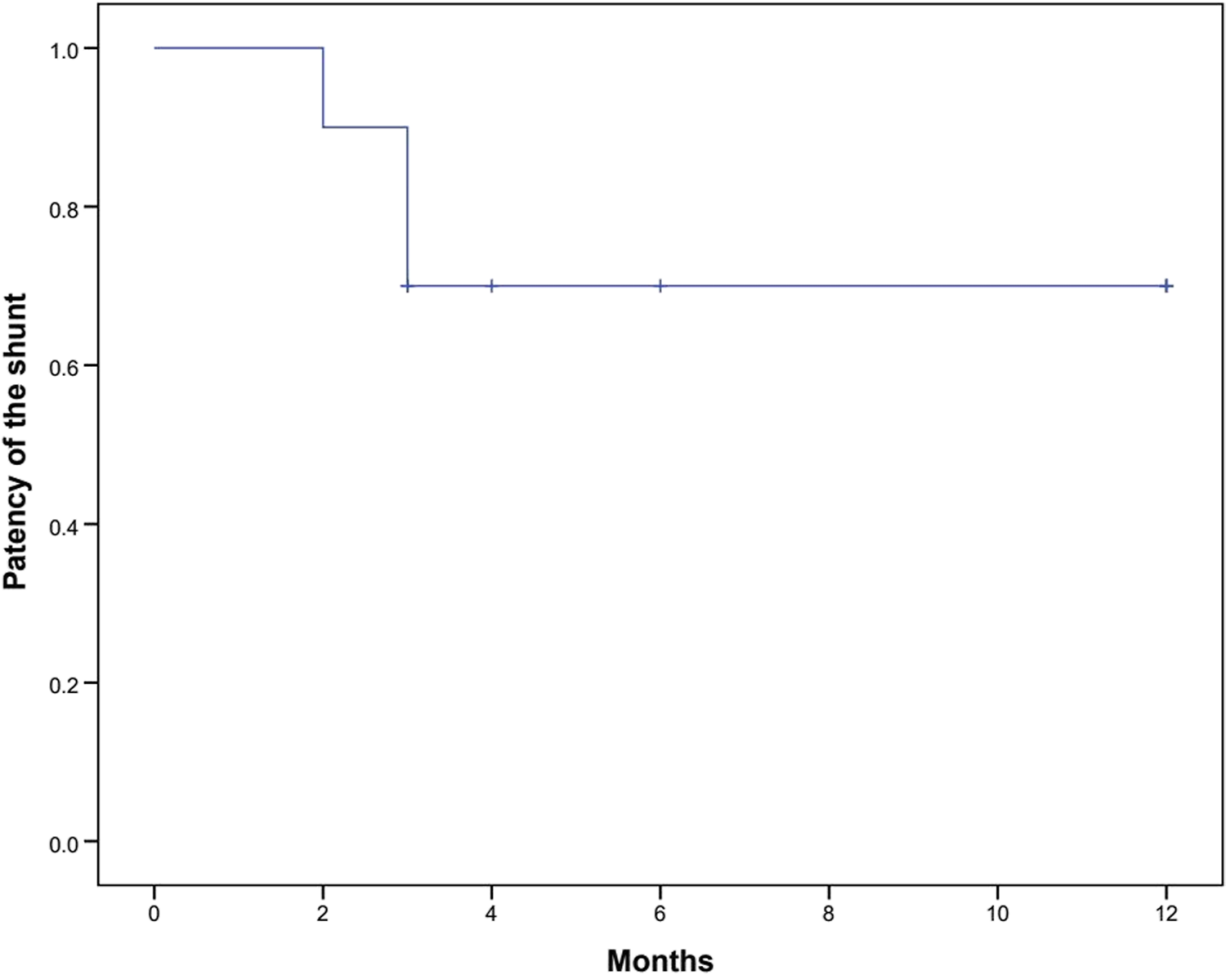
Mean platelet counts before and 1, 2, and 4 weeks after the operation

Shunt patency was confirmed in all patients during the first postoperative month (Fig. 3). However, the SRS closed in four patients during the follow-up period (long-term shunt patency rate = 60%). Shunt occlusion occurred in one patient 2 months after surgery, in two patients 3 months, and in one patient 13 months after surgery (Fig. 4). However, during long-term follow-up, none of the patients developed signs and complications of left-sided portal hypertension such as gastrointestinal bleeding or splenomegaly. One patient (10%) had thrombocytopenia before the operation, and six patients (60%) had thrombocytopenia within 1 week after the operation. However, the platelet count returned to normal within 4 weeks (Fig. 5). There was no significant difference in splenic volume before and after SRS (Table 2, *p* = 0.649), indicating that there was no development of left-sided portal hypertension in the patients with SRS.

## Discussion

In this study, we describe our initial experience of reconstructing the gastric and splenic venous drainage via SRS during a pancreatoduodenectomy. The reported outcomes in our cohort of ten patients with locally advanced pancreatic cancer reveal remarkable technical and intraoperative advantages of this technique, including 100% technical success rate and negligible additional operating time. No patients developed procedure-related morbidities during postoperative follow-up, and only one patient died from non-SRS-related complications during the postoperative hospital stay. Furthermore, no patients showed signs of left-sided portal hypertension. All patients had short-term shunt patency, and this patency remained in 60% of patients. However, no signs of left-sided portal hypertension were detected in patients with shunt closure. In all patients, major collaterals of the gastric venous route, such as coronary vein and IMV, were divided. Therefore, we hypothesize that the SRS closed due to low blood flow, as the SRS was not physiologically necessary in these patients. These findings suggest that SRS is a safe, feasible, and effective procedure during pancreatoduodenectomy, with encouraging results.

Pancreatoduodenectomy with a tumor-free resection margin is one of the most vital factors that lead to significantly better long-term survival in patients with pancreatic cancer [25–27]. In these cases, extended pancreatoduodenectomy with vascular reconstruction is needed to achieve a complete tumor resection [4, 20, 28, 29]. During oncological pancreatoduodenectomy, segmental resection of the SMV-PV-SV confluence may be necessary if the tumor is close to or has infiltrated. SV ligation is a principle component of this procedure. SV drainage may occur through the IMV if it merges into the SV of the left side of the resection plane [30]. If this route does not exist, the splenocolic collateral or the gastric venous route is the only possible way to drain venous blood flow from the spleen [16]. This increases the flow through the gastric and esophageal veins resulting in left-sided portal hypertension that may result in gastrointestinal hemorrhage [10].

Impaired gastric venous drainage, especially in the cases of total pancreatectomy and resection of the gastric venous route due to tumor infiltration, may cause gastric venous congestion, which should be treated through partial gastrectomy. It has been shown that the patients undergoing additional gastric resection faced with significantly deteriorated postoperative nutritional status and quality of life [31]. Therefore, surgical reconstructive venous drainage of the gastric and splenic vein may provide an alternative to avoid venous congestion and additional gastrectomy [18, 32].

Several studies have described different surgical and reconstructive techniques and venous flow patterns to prevent the harmful effects of portal confluence resection during pancreatoduodenectomy [17, 33–35]. In previous reports, authors have opposed the efficiency of SV reconstruction by emphasizing the lack of complications after pancreatoduodenectomy with SV ligation [36, 37]. Several alternative venous routes and drainage flows have been described which were believed to prevent left-sided portal hypertension and subsequent complications [10]. In a survey of five patients, collateral venous pattern progression was evaluated after SV ligation [6]. No patients developed splenomegaly or other venous complications induced by SV occlusion during a follow-up period of 6 to 8 months. The draining veins developed in these patients were similar to splenocolic collaterals, and no varicose changes were observed. SV ligation was not associated with any complications, particularly in patients whose left gastric vein was preserved [33, 38]. These findings suggest that preservation of the left gastric vein, or the middle colic vein, may prevent left-sided portal hypertension after SV ligation [10]. These veins provide several options for venous drainage, including through the venous arc of Barkow (blood drainage from left gastroepiploic vein into the left epiploic vein [39]) and middle colic vein to the SMV and through short and left gastric veins into the PV. However, in patients with pancreatic head cancer, portal confluence resection is inevitable in case of confluence involvement during pancreatoduodenectomy [40, 41]. This means that ligation of major venous contributors, such as the gastrocolic trunk of Henle, left/right gastric vein, and occasionally the IMV is required [42]. The middle colic and gastrocolic veins may be divided during resection depending on tumor location and extent, but both do usually not contribute to gastric venous drainage and, therefore, have no impact on the decision making for SRS.

As shown by our data, locally advanced pancreatic tumors can be safely resected with an SRS to achieve a stable splenic volume during the immediate postoperative period. SRS may be an alternative method to preserve gastric venous drainage and avoid a near-total gastrectomy and thereby improve the patients’ quality of life [16]. Some authors have suggested restricting SRS to patients with direct drainage from the IMV to the SMV. However, others believe that SRS should be considered in patients with sacrificed gastric venous drainage (left gastric vein) during pancreatoduodenectomy with venous resection and a pancreaticogastrostomy. Some studies have shown superior postoperative outcomes following SRS than following SV ligation [18]. Based on our experience, performing SRS is not essential for all patients undergoing pancreatoduodenectomy with portal confluence resection, but it should be considered in patients with a sign of intraoperative venous congestion. Vascular reconstruction through an SRS is technically straightforward and provides outstanding postoperative outcomes.

In addition to illustrating the surgical techniques, this study aimed to analyze and report surgical outcomes, such as splenic volume changes and patency of the shunt after SRS. However, the study is limited by small sample size and short follow-up period. To better understand the changes after SRS as well as its effectivity, these findings should be assessed in future large-scale prospective or randomized controlled trials.

## Conclusion

SRS produces encouraging and reliable outcomes without procedure-specific complications in splenic and stomach drainage after pancreatoduodenectomy with portal confluence resection. Based on our experience, there is no need to perform SRS in patients where gastric venous drainage is preserved. However, SRS may improve postoperative outcomes in cases of portal venous confluence resection with a sign of intraoperative gastric venous congestion.

## Data Availability

All data for this study are available as part of the article, and no additional source data were required.
